# Supplementary material for: Gut Microbiota and Metabolites in Atrial Fibrillation Patients and Their Changes after Catheter Ablation
Source: Microbiol Spectr. 2022 Apr 6;10(2):e01077-21. doi: 10.1128/spectrum.01077-21 (PMC9045169; doi:10.1128/spectrum.01077-21)
Supplement: SUPPLEMENTAL FILE 1 — Supplemental material. Download SPECTRUM01077-21_Supp_1_seq10.pdf, PDF file, 6.1 MB [file spectrum01077-21_supp_1_seq10.pdf]

Table S1 Screening results of differential metabolites

Compared Samples	Total Ident.	Total Sig.	Sig.Up	Sig.down
AF2.vs.AF1_pos	1453	71	16	55
AF2.vs.AF1_neg	691	31	12	19
AF2.vs.AF1_pos	1453	71	16	55
AF2.vs.AF1_neg	691	31	12	19

Compared Samples：Comparare sample pairs. Total Ident.：Total identification results of the metabolites. Total Sig.：Total number of metabolites with significant differences. Sig.Up：Significant upregulated metabolites. Sig.down：Significant downregulated metabolites.

Table S2 Comparison of clinical data of metabolic cohort studies

Characteristic	AF	Control	P-value
Number	8	10	/
Age/years	52.63±9.27	45.10±7.94	0.082
Male/female Sex	6/2	7/3	1.00
Body mass index	23.90±2.21	24.30±3.06	0.762
HTN	2	4	0.608
T2DM	0	0	/
Total Cholesterol	4.30±1.38	4.99±0.58	0.214
Triglyceride	1.32（1.01，1.79）	1.53（1.32，3.16）	0.131
LDL	2.91±1.00	3.22±0.50	0.446
Creatinine	81.00±17.27	70.70±14.16	0.183
Uric acid	338.50±37.69	386.00±28.69	0.322
Total bilirubin	12.11±4.85	13.60±6.76	0.608
ALT	14.50（12.25，19.00）	17.50 （9.50，19.25）	0.824
Drug use			
ARB / ACEI	2	2	1.00
β receptor blockers	4	1	0.118
Ca^{2+} channel blocker	1	1	1.00

Diuretic	1	0	0.444
Statins	4	2	0.321
Ezetimibe	0	2	0.477
Aspirin	1	2	1.00
PPI	1	1	1.00
Amiodarone	0	0	/
Oral Anticoagulation therapy	6	0	0.002

Data are presented as mean ± standard deviation or median (interquartile range), as appropriate. HTN: Hypertension; T2DM: Type 2 diabetes; LDL: Low densith lipoprotein; ALT: Alanine Aminotransferase; ARB: Angiotensin receptor antagonist; ACEI: Angiotensin converting enzyme inhibitor

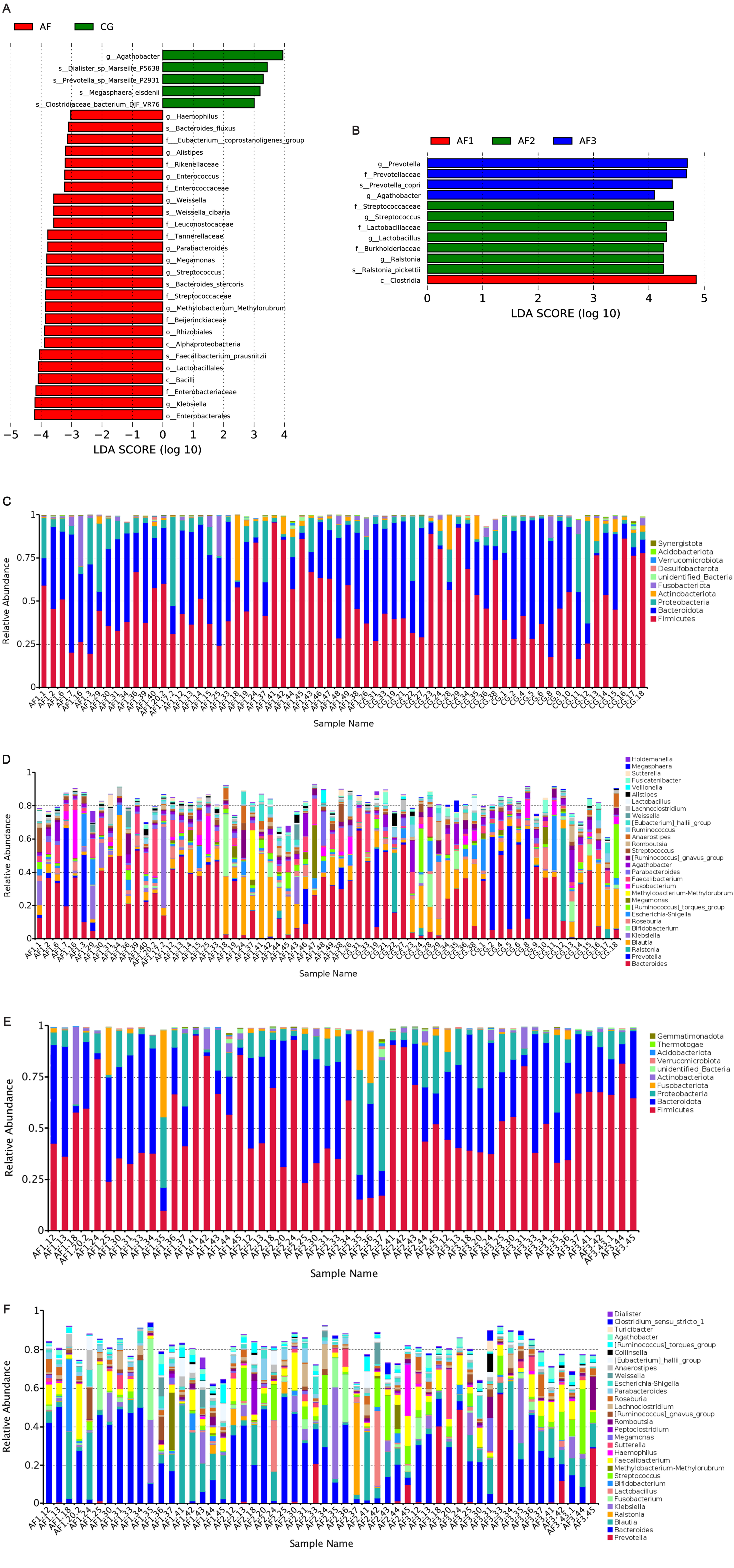

Supplementary Figure. The relative abundance of species for all individuals.

A, LEfSe analysis between AF patients and CG. B, LEfSe analysis of AF patients before and after catheter ablation.

C and D, The relative species abundance of AF patients and control subjects (E, Top 10 at the phylum level; F, Top 30 at the genus level).

E and F, The relative species abundance of AF patients before and after catheter ablation (G, Top 10 at the phylum level; H, Top 30 at the genus level).
